# Letter to the Editor on Previously Published GPHF-Minilab Assessment

**DOI:** 10.4269/ajtmh.18-0127a

**Published:** 2018-06

**Authors:** Richard Jähnke

**Affiliations:** Global Pharma Health Fund (GPHF); Project Office; Frankfurt, Germany; E-mail: richard.jaehnke@gphf.org

Dear Sir,

This is to clarify some points presented in the paper “Diagnostic Accuracy of Global Pharma Health Fund (GPHF) Minilab™ in Assessing Pharmacopoeial Quality of Antimicrobials.”^1^ The International Institute of Research against Counterfeit Medicines identified China as one of the major sources of poor-quality antibacterial medicines.^2^ This is why the publication is concerned with the quality of antibacterial medicines in China and compares high and low technology options for an in-country medicine quality monitoring system. In this comparison, high-pressure liquid chromatography (HPLC) serves as a high-tech standard and the Minilab of the GPHF, a charity run by Merck in Germany, as a low-tech standard. The GPHF Minilab is a field test device developed for rapid quality verification of priority medicines, for example anti-infective medicines, and the detection of fake and substandard pharmaceuticals.^3^ The authors of the article imply that the Minilab, a self-contained portable mini-laboratory specifically developed for use in low-income countries, was intended to replace HPLC for the assessment of drugs, which was never the intent of the developers of this system. Rather, the Minilab is intended to allow resource constrained settings to boost their testing capacities when addressing the proliferation of falsified and substandard medicines with wrong, low, or zero drug content. For this purpose, testing the absence of an active pharmaceutical ingredient does not necessarily require advanced and costly HPLC.

Basic screening technologies will help in lowering the costs of assessment of pharmaceuticals. Screening can start with a simple physical inspection for gross and obvious quality defects, for example, particulate matter in an injectable and a passed expiry date on the label. It can include measurement of tablet and capsule mass to identify variations. Simplified disintegration tests can predict improper drug release due to poor tablet and capsule formulation. As a next step, the Minilab employs thin layer chromatography for drug identity testing and semi-quantitative assay readings (±10%). The costs of Minilab procurement, use, and maintenance are low, and the system requires minimal training. It is an entry-level technology never intended as a laboratory or HPLC replacement. In sub-Saharan Africa and Southeast Asia, the GPHF Minilab was very successful in the detection of fake antimalarial medicines with no or little active pharmaceutical ingredients.^4^ Considering this context, in the recent report from China, the Minilab detected most poor-quality drugs in the study. A combination of both HPLC and Minilab could reduce costs and maintain comprehensive, quality surveillance as proofed by the Promoting the Quality of Medicines program of the U.S. Pharmacopeial Convention in Africa and Asia.^4^ In the past 10 years, more than 1,000 poor-quality medicines failed Minilab screening.^5^ Hence, Minilabs help in saving lives. Thus, the GPHF Minilab has an important role to play, but it should not be seen as a replacement for HPLC for the formal evaluation of pharmaceuticals.
